# Pedestrian Pose Recognition Based on Frequency-Modulated Continuous-Wave Radar with Meta-Learning

**DOI:** 10.3390/s24092932

**Published:** 2024-05-05

**Authors:** Jiajia Shi, Qiang Zhang, Quan Shi, Liu Chu, Robin Braun

**Affiliations:** 1 School of Transportation and Civil Engineering, Nantong University, Nantong 226001, China; shijj@ntu.edu.cn (J.S.); 2118320012@stmail.ntu.edu.cn (Q.Z.); 2 Center for Transformative Science, ShanghaiTech University, Shanghai 201210, China; chuliu@shanghaitech.edu.cn; 3 Faculty of Engineering and Information Technology, University of Technology Sydney, Sydney, NSW 2050, Australia; robin.braun@uts.edu.au

**Keywords:** millimeter-wave radar, pose recognition, micro-Doppler, channel attention mechanism, angular margin loss function, MAML

## Abstract

With the continuous advancement of autonomous driving and monitoring technologies, there is increasing attention on non-intrusive target monitoring and recognition. This paper proposes an ArcFace SE-attention model-agnostic meta-learning approach (AS-MAML) by integrating attention mechanisms into residual networks for pedestrian gait recognition using frequency-modulated continuous-wave (FMCW) millimeter-wave radar through meta-learning. We enhance the feature extraction capability of the base network using channel attention mechanisms and integrate the additive angular margin loss function (ArcFace loss) into the inner loop of MAML to constrain inner loop optimization and improve radar discrimination. Then, this network is used to classify small-sample micro-Doppler images obtained from millimeter-wave radar as the data source for pose recognition. Experimental tests were conducted on pose estimation and image classification tasks. The results demonstrate significant detection and recognition performance, with an accuracy of 94.5%, accompanied by a 95% confidence interval. Additionally, on the open-source dataset DIAT-*μ*RadHAR, which is specially processed to increase classification difficulty, the network achieves a classification accuracy of 85.9%.

## 1. Introduction

Human activity recognition technology has received widespread attention recently, with the growing demand for intelligent health monitoring, traffic safety, and traffic management. Different application scenarios have placed higher demands on the accuracy and sensitivity of activity recognition. In short-distance human-monitoring environments, the main sensors used are divided into two categories: wearable and non-wearable sensors. Wearable sensors are typically attached to a specific part of the body through buttons, straps, or placed in pockets, while some use pressure sensors placed inside shoes for human activity recognition. These sensors are capable of capturing high-resolution data from human activities and representing them in forms such as acceleration, angular velocity, speed, and displacement. However, these methods often have strong invasiveness, may involve personal privacy concerns, are inconvenient to wear, and require cooperation from pedestrians.

In the field of non-wearable sensors, video, radio frequency (RF), and radar technology have become the hot spots for human activity recognition. The video-based scheme is relatively mature, and video-based identification is a common measure at this stage. However, the video-based method is objectively vulnerable to environmental interference; for instance, light, rain, snow, and other conditions can affect the accuracy of video detection. Subjectively, video-based schemes invade the living environment of detection targets and infringe on their privacy. Therefore, in the field of human body recognition, RF and radar-based methods have become popular under the condition of privacy protection [1]. The RF-based approach can obtain high recognition accuracy in an indoor environment. In [2], a small sample experiment is conducted on the RF dataset collected indoors, and the accuracy rate is 94.49%, indicating that the RF-based approach can effectively identify the behavior of the target in an indoor environment. However, due to a number of factors, in outdoor environments, the RF mode of performance is not as good as millimeter-wave radar. First, Wi-Fi in the same frequency band is relatively crowded and easy to interfere with each other. Second, the main purpose of Wi-Fi is to transmit data, which needs to transmit a large amount of information to the carrier. Therefore, the detection performance and anti-interference ability of the target are far lower than that of the radar specially used for detection, and the detection accuracy is limited to the environment set by itself. Third, Wi-Fi generally has omnidirectional antennas, while radar antennas are directional and can detect targets in a specific area without interference from targets in other directions. Therefore, to compare Wi-Fi and radar, radar’s performance is significantly better than Wi-Fi in the outdoor environment.

Radar captures human activity features in complex environments through its Doppler signals, enabling the acquisition of target information even under special weather conditions or certain occlusion scenarios [3]. When a pedestrian moves, the relative motion between the body parts and the radar sensor causes a Doppler effect. This effect causes a change in the frequency of the reflected wave received by the radar, which contains dynamic information about the pedestrian’s gait. The Doppler frequency is the specific parameter that describes this frequency change. The frequency variation is closely related to the pedestrian’s walking speed, step frequency, and gait characteristics. By analyzing the Doppler frequency, the features related to pedestrian gait can be extracted. When observing pedestrians on a finer scale, the movements of their body parts produce small Doppler frequency changes, which are called micro-Doppler effects. Therefore, human activities can be accurately represented by analyzing the micro-Doppler characteristics of echo signals [4].

In recent years, neural networks have become a tool for pedestrian pose recognition. Previous researchers have employed various machine learning techniques such as principal component analysis, multilayer perceptron, and support vector machines [5]. However, these methods primarily focus on classification algorithms with limited efficiency in feature extraction [6]. Consequently, deep learning has become the mainstream tool due to its ability to automatically extract features, self-adjust, and self-regulate [7,8,9]. Ajay Waghumbare et al. [10] utilized different pre-trained deep convolutional neural network (DCNNs) models, such as VGG-16, VGG-19, and Inception V3, and fine-tuned these models to effectively perform recognition of human activity on the DIAT-μRadHAR human activity dataset. Ibrahim Alnujaim et al. [11] trained a DCNN using micro-Doppler features generated by generative adversarial networks (GANs) as well as raw data, resulting in improved classification accuracy. Furthermore, with the recent popularity of attention mechanisms, many methods have incorporated attention mechanisms to extract features and have achieved corresponding successes. Fahad Jibrin Abdu et al. [12] designed an elderly fall detection system using radar signal micro-Doppler features extracted by a convolutional neural network (CNN) and by proposing a channel attention network using canonical correlation analysis (CCA) algorithm, effectively fused the extracted features for radar data recognition.

Considering the timeliness and generalization required for collecting data in real-world environments, in this paper, we propose the use of meta-learning strategies to achieve higher training accuracy with small sample sizes and improve the network’s generalization ability. We focus on researching and optimizing the model-agnostic meta-learning (MAML) framework in the context of human activity recognition to enhance precision. Specifically, through attention mechanisms, we enhance the network’s ability to capture key information, making the inner loop of MAML more compact. Additionally, by employing the ArcFace loss function to constrain the inner loop, we ensure the model has good inter-class discrimination and intra-class cohesion.

The contribution of this paper is as follows:

(1) We propose a framework that integrates the attention mechanism into residual networks and optimizes the initialization parameters through MAML to recognize pedestrian movements captured by FMCW radar.
(2) In this article, we employ the ArcFace loss function to constrain the inner loop that forms the AS-MAML network, aiming to enhance inter-class diversity and discriminability towards intra-class variations.
(3) By validating open-source datasets such as DIAT-*μ*RadHAR, we ensure the effectiveness of our network.

The first section of this paper mainly describes the application of FMCW millimeter-wave radar in the field of gait recognition, as well as the main contribution of the article. Section 2 introduces relevant research in the field of millimeter-wave radar gait recognition, as well as some current research status of small-sample gait learning. Section 3 describes the relevant calculations of the FMCW radar, which mainly includes radar signal processing, micro-Doppler effect calculation, and signal noise reduction processing. Section 4 introduces the proposed network and the methods used to optimize the network through the channel attention mechanism and ArcFace loss function. Section 5 is the experimental part, which compares the performance of the proposed network with other networks through various experimental analyses and verifies the methods used on open-source datasets. Section 6 is the summary.

## 2. Related Work

In recent years, with the high integration of modern radar, i.e., the gradual miniaturization of radar systems, civilian radar has entered all aspects of people’s lives. Over the years, researchers have made significant progress in millimeter-wave radar human behavior recognition. Papadopoulos, K et al. [13] conducted a comparative study on the current research progress of radar human activity recognition based on machine learning. He believes that the currently used machine learning methods mainly include support vector machines (SVMs), convolutional neural networks, recurrent neural networks (RNNs), long short-term memory (LSTM) neural networks, stacked autoencoders, convolutional autoencoders, and transformers.

Some traditional methods, such as SVMs and principal component analysis (PCA), are used for the initial recognition of pedestrian gait data. In [14], SVMs were used to classify seven different human activities measured by ultra-wideband radar: walking, running, rotating, punching, crawling, standing still, and the transition between sitting and standing. Classification was achieved using the time variations of the returned signals from human objects. Features were captured by PCA. SVMs were proposed as the classifier. When capturing features using principal component analysis, the most significant 30 component coefficients were retained, reducing the overall data size by 98.7% while still retaining 95% of the signature information. In the final SVM classification and recognition, due to the existence of inherently similar actions, the average classification accuracy was only 89.88%.

In [15], researchers compared SVMs and CNNs on the Harth and Har70+ datasets, weighing the model performance and resource consumption (i.e., hardware-optimized model comparison) to provide information for FPGA implementation. After comparison, the F1 score of the unoptimized CNN model was 7.7% higher than that of the unoptimized SVM model, and the resource utilization was improved by two to three times. Currently, CNNs, RNNs, and LSTM neural networks are commonly used classification algorithms. Since its introduction by Yann LeCun in the 1980s, CNNs have received significant attention in the scientific community and have become increasingly important in signal processing. 

Because gait data are presented over time, networks designed for handling temporal data, such as LSTM neural networks, are used for gait data classification. Often, LSTM can be combined with other networks. In [16], a combination of CNNs and LSTM neural networks was used. After obtaining features through a one-dimensional CNN, the features were integrated into an LSTM neural network with attention mechanisms as a time series to achieve human activity recognition. The aim was to achieve a lighter network and achieved a recognition accuracy of 96.9%.

Gait data can also be represented in the form of spectrograms, and micro-Doppler images can vividly present the characteristics of gait data. Therefore, CNNs are also a hot research topic in this field. In [17], continuous-wave (CW) K-24 GHz band radar sensors were used to collect signals, and the collected radar motion data were classified into three main behaviors: non-human motion, human walking, and human walking without arm swinging. The collected signals were processed using STFT, Mel spectrograms, and Mel-frequency cepstral coefficients. The latter two methods are commonly used in audio processing but were used here to obtain the micro-Doppler spectrograms of all motion data. The processed data were then input into a simplified 2D CNN architecture for feature extraction and classification. The network trained on Mel-scale frequency cepstral coefficients (MFCCs) features achieved a classification accuracy of 97.93% in the final experimental results.

These neural networks can achieve relatively high classification accuracy, which is achieved based on a large amount of data. Currently, there is a lot of research in this area, and by using mature datasets or collecting more data, the networks can adapt to the corresponding training results [18,19,20,21]. However, in practical application environments, considering the difficulties in data collection, most of the available training data is relatively limited. Therefore, we need to take corresponding measures to alleviate the impact of insufficient data. Some researchers have also considered the situation of limited datasets and used GANs to expand the dataset to improve accuracy. Alnujaim et al. [22] proposed the generation of synthetic micro-Doppler features from different angles using conditional generative adversarial networks. They believe that the synthesis of micro-Doppler signals is an alternative to collecting a large amount of human activity data. By synthesizing micro-Doppler features of human activities from different radar perspectives with input data from a single perspective, they studied the feasibility of data augmentation by examining multi-target micro-Doppler features with incremental angles of 45 degrees. Finally, conditional generative adversarial networks (CGANs) were used to synthesize micro-Doppler features at specific angles.

## 3. Micro-Doppler Signal Processing

### 3.1. Radar Signal Analysis

**Figure 1:**
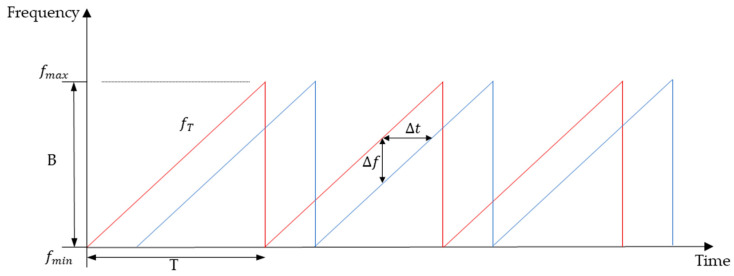
Time-related characteristics of the sawtooth modulation signal. The red represents the transmitting signal, and the blue represents the receiving signal.

In the identification of human activities, FMCW radar is more suitable for human perception than continuous wave radar, as it can measure speed and distance at the same time with high sensitivity and high anti-interference ability. FMCW radar emits continuous electromagnetic waves with constant amplitude and modulated frequencies and processes the electromagnetic waves reflected by the targets. The FMCW radar system generates sinusoidal amplified RF signals through high-frequency oscillation units, and the frequency shows a sawtooth shape within the duration, as shown in Figure 1.

(1) $$f_{max}-f_{min}=\frac{∆f}{∆t}\cdot T$$

Radar emits a series of chirp signals with a period of *T* and a bandwidth of *B*. Each chirp can be represented as:

(2) $$s_{t}\left(t\right)=Aexp\left(j2\pi \left(f_{0}t+\frac{1}{2}\frac{B}{T}t^{2}\right)\right)$$

where *A* is the amplitude of the transmitted signal, and $f_{0}$ is the carrier frequency.

When the transmitted signal detects the target, an echo signal is generated. The received echo signal and the transmitted signal mix together to generate an intermediate frequency (IF) signal:

(3) $$s_{IF}\left(t\right)=KAexp\left(j\pi \left(f_{0}t_{d}+2\frac{B}{T}t_{d}t-\frac{B}{T}{t_{d}}^{2}\right)\right)$$

where K represents gain and $t_{d}$ is the delay time for the target arrival.

At this point, the Doppler frequency shift caused by target motion is given by $f_{D}=-\frac{2vf_{T}}{c}$, where $f_{T}$ is the frequency of the transmitted signal. When the target is a human body, the movement of the human torso constitutes the main component of the Doppler signal in the echo, while the limbs appear as micro-motion signals along the edges of the main Doppler signal. This is a characteristic introduced by the human body in the micro-Doppler signal. We need the micro-Doppler signal of human micro-motion to conduct further identification research.

### 3.2. Micro-Motion Signal Processing

**Figure 2:**
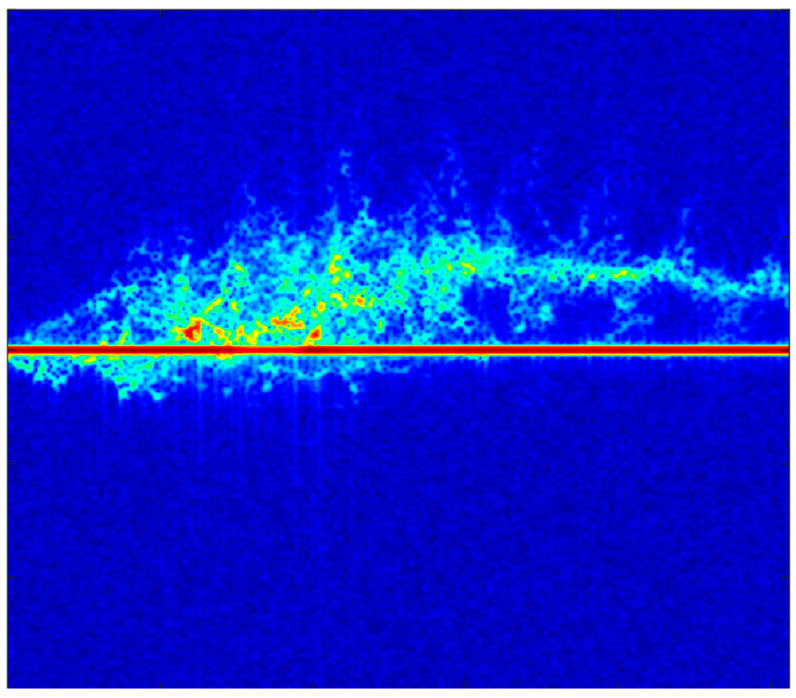
Micro-Doppler image of a walking person.

When the torso causes Doppler frequency shift to a certain extent, the limbs generate oscillatory sidebands, which are known as micro-Doppler signals [23]. These micro-Doppler signals appear in a resolvable form in the time–frequency plane, enabling the classification and recognition tasks of micro-Doppler images in deep learning. Figure 2 shows a micro-Doppler image of a walking person.

Micro-Doppler signals can be obtained through time-correlated frequency domain transformations. First, the raw data of the signal is converted into a time-dependent distance distribution through a fast Fourier transform. Then, the time-dependent spectral distribution of the signal is calculated through the short-time Fourier transform. Window function operations are performed on a joint time–frequency platform, defined as the sum of signal values multiplied by a window function, which is typically a Gaussian function. The Doppler sequence generated by each sliding window is arranged in slow time to form a TF image. At this time, the spectrum can be expressed as:

(4) $$X\left(\tau ,f\right)=\int_{-\infty }^{\infty }x\left(t\right)\omega \left(t-\tau \right)e^{-j2\pi ft}dt$$

where $X\left(\tau ,f\right)$ is the time–frequency domain representation at time m and frequency, *f*$\omega \left(t-\tau \right)$ is the time-shifted version of the window function at time, and τ. $x\left(t\right)$ is a continuous-time signal.

Choose a window function with window length T and a time step between windows D. In the time-discrete domain, the time-discrete STFT applied to the time-range distribution matrix can be obtained using the following formula:

(5) $$X\left[n,k\right]=\sum_{n=-\infty }^{\infty }x\left[m\right]\omega \left(m-nD\right)e^{-j2\pi km/N}$$

where $X\left[n,k\right]$ refers to the discrete time–frequency domain representation at time point *n* and frequency point *k*.$x\left[n\right]$ represents the sample of signal $x\left(t\right)$ at time point *n*T. $\omega \left(m-nD\right)$ is the time-shifted version of the window function at time *n.* N is the length of the FFT.

**Figure 3:**
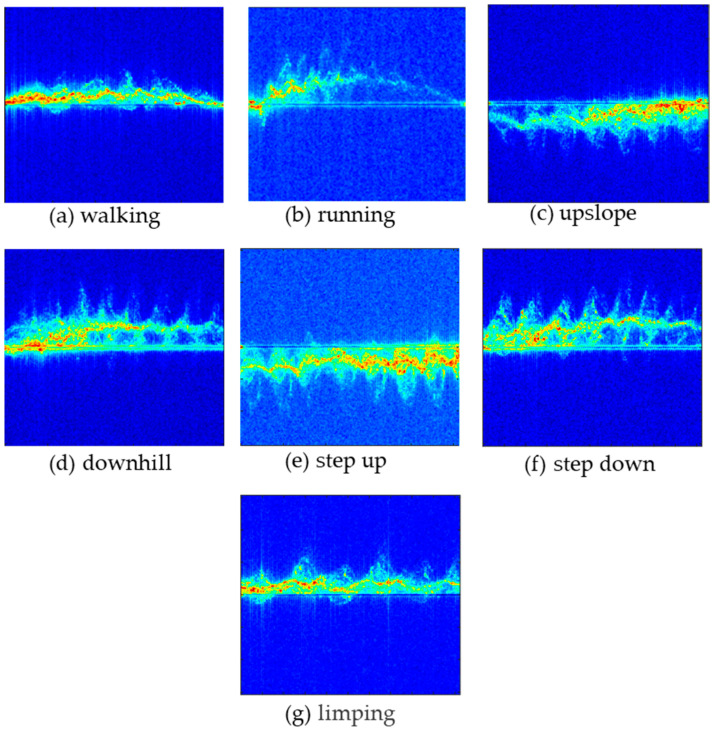
Micro-Doppler images of seven gait postures.

In practical applications and subsequent open-environment experimental collections, the micro-motion power introduced by human activities is very small and can be easily affected by strong static clutter. As shown in the middle red line in Figure 2, this is the interference of static targets on the image in the actual collection environment. When the micro-Doppler image is normalized, these clutters can affect the quality of the collected micro-Doppler image, making it inconvenient for subsequent research. Therefore, it is necessary to suppress the obtained micro-Doppler image using an MTI frequency filter to remove the effect of clutter. Additionally, a Butterworth high-pass filter is used to process the signal, preserving effective frequencies and making the micro-Doppler image more observable. The Butterworth high-pass filter can be represented as:

(6) $$H\left(j\omega \right)=\frac{1}{\sqrt{1+{\left(\frac{\omega }{\omega_{c}}\right)}^{2n}}}$$

where $H\left(j\omega \right)$ is the transfer function of the filter, ω is the angular frequency, $\omega_{c}$ is the cutoff frequency, and n is the order of the filter. By solving the modulus of the transfer function, the gain characteristics of the filter can be obtained. In practical use, we set n = 4 and $\omega_{c}$ = 0.0075. Figure 3 shows seven types of motion signals after clutter suppression.

When obtaining Doppler images, the first step is to employ window functions to apply windowing to the collected data, reducing spectral leakage. Subsequently, the windowed data is subjected to a short-time Fourier transform (STFT) to generate a spectrogram. By analyzing the spectrogram, one can derive a time-velocity Doppler image that represents the target’s velocity changes.

The Moving Target Indication (MTI) filter is primarily used in radar signal processing for detecting and tracking moving targets. In the MTI filter, there is a time interval between two consecutive radar pulses. By performing a time-domain differencing operation on the received signal between these two consecutive pulses, it is possible to eliminate the signal components of stationary targets. Additionally, a low-pass filter is employed within the filter to remove high-frequency components, further suppressing stationary or slow-moving clutter.

Then, anisotropic denoising will be applied to the micro-Doppler images. Anisotropic filtering can effectively preserve characteristics such as gait outlines and motion details while reducing noise.

Following data preprocessing, we simply compare the training datasets before and after denoising. The accuracy of the training before denoising is 83.14%, whereas after denoising, it improves to 94.3%. This indicates that the noise in the original image is still quite noticeable.

## 4. Improved Meta-Learning

**Figure 4:**
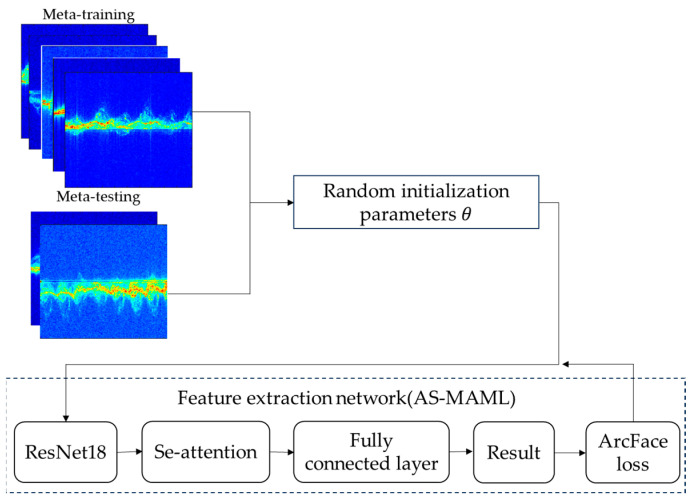
AS-MAML network structure diagram.

The AS-MAML network structure diagram is shown in Figure 4.

The main network we use is Resnet18, where the outputs of hidden layers in the network are directly added to distant, hidden layers via residual and skip connections. This approach alleviates the vanishing gradient problem and makes the network easier to train and optimize.

When facing the problem of low feature extraction rate and low inter-class discrimination, we adopt channel attention and additional angular margin loss to optimize the network. This enhances the performance and inter-class discrimination of the network.

### 4.1. Model-Agnostic Meta-Learning (MAML)

Meta-learning, also known as “learning to learn”, aims to acquire a “learning method” by training on multiple related tasks, enabling this method to quickly learn and adapt to new tasks [24]. Due to the inherent strong transferability and generalization ability of meta-learning, its application scope includes but is not limited to small sample learning, transfer learning, reinforcement learning, meta-reinforcement learning, and adaptive learning.

In the context of transfer learning, a meta-learning approach enables the model to use the knowledge and experience gained in the source domain to accelerate the learning process in the target domain. In reinforcement learning scenarios, meta-learning methods help to design learning strategies and fine-tune algorithm parameters so that they can adapt to different environments and tasks. Specifically, meta-reinforcement learning needs to acquire learning strategies in various reinforcement learning tasks to improve the performance of the learning algorithm on unknown tasks. The application of meta-learning techniques in this field enables the design of meta-reinforcement learning algorithms to facilitate knowledge transfer and generalization across different tasks. Finally, in the face of a dynamic environment or changing data distribution, meta-learning methods promote the adaptive adjustment of learning strategies and model parameters so that the model can effectively adapt to new situations. Of course, meta-learning networks specially designed for small samples have their advantages in small sample tasks, which can obtain the highest possible generalization performance and recognition accuracy through the smallest sample size.

While a substantial amount of suitable data can be obtained through sampling in indoor environments, data collection can be more challenging in outdoor scenarios, resulting in smaller datasets. In the context of small-sample multi-classification tasks, meta-learning can enhance the performance of models in few-shot learning by learning shared feature representations across tasks or acquiring adaptive optimization strategies.

**Figure 5:**
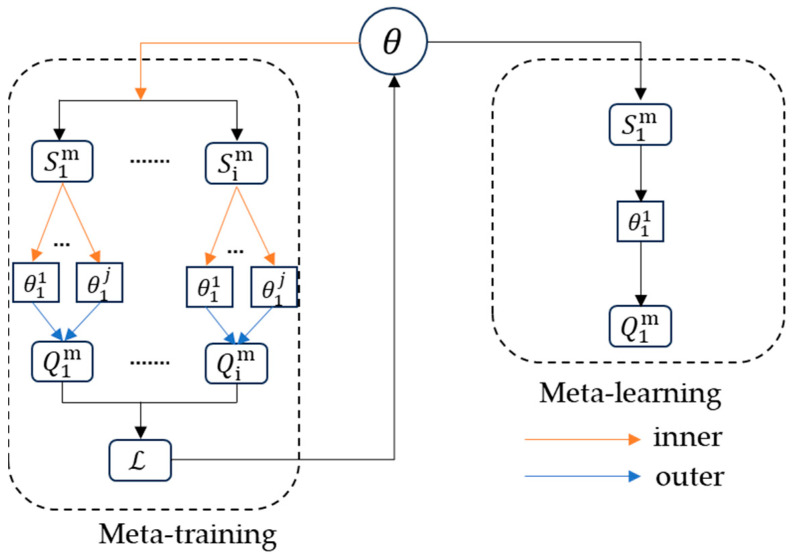
MAML update processing.

MAML is a popular framework in meta-learning, aiming to discover an optimal initialization of model parameters that enables the model to adapt to various tasks with only a few gradient updates. MAML can also be defined as a two-level optimization problem: inner loop optimization and outer loop optimization. In the inner loop optimization, each task $T_{i}^{m}=\left\{S_{i}^{m},Q_{i}^{m}\right\}$ in the support set $S_{i}^{m}$ is used to fine-tune the initialization of model parameters through a fixed number of gradient descent steps. As shown in Figure 5, MAML usually uses an inner and outer loop update to obtain common parameters on the task, helping the model to better generalize. MAML typically employs the cross-entropy loss function for update learning:

(7) $$\theta_{i,j+1}=\theta_{i,j}-\beta_{inner}\nabla_{\theta }L\left(f_{\theta_{i,j}};S_{i}^{m}\right)$$

where j refers to the internal update steps, $\beta_{inner}$ is the internal learning rate of the weights, $f_{i,j}$ represents a parameterized function with parameter $\vartheta_{i,j}$, and L signifies the loss function; the final weight of the base learner after J updates is $\theta^{\prime }$=$\vartheta_{i,J}$. After M iterations of gradient descent, the task of sampling from the query set $Q_{i}^{m}$ updates the meta-learner according to the following rules:

(8) $$\theta \leftarrow \theta -\beta_{meta}\sum_{i=1}^{B}L\left(f_{\theta^{\prime }};Q_{i}^{m}\right)$$

where *B* refers to a set of tasks encompassed within a batch, and $\beta_{meta}$ signifies the learning rate in outer loop optimization.

### 4.2. Attention Mechanism

The mechanism of channel attention aims to enhance the model’s processing of specific signals in the input data. By automatically focusing on the most important channels in the input signal, SE-attention achieves better encoding and extraction of information. The channel attention mechanism can be applied to various types of network structures and tasks, including image classification, object detection, and segmentation, making it one of the important tools commonly used in modern deep learning [25].

In the residual network, we introduced a channel attention mechanism in the final stage of each network to capture the relationship between global features. During the training on the testing dataset, the network without attention mechanism exhibited overfitting.

Let the input feature map $x^{f}\in r^{h\times w\times c}$, where c is the number of channels in the input feature map, h and w are the height and width of the feature map, respectively. The channel attention mechanism calculates the weight of each channel and scales each channel in the input feature map based on its weight.

First, we obtain the average value $\alpha_{c}$ of the entire feature map through the global average pooling layer, where *c* represents the channel dimension:

(9) $$\alpha_{c}=\frac{1}{h\times w}\sum_{i=1}^{h}\sum_{j=1}^{w}{x^{f}}_{ijc}$$

Subsequently, we employ two fully connected layers to generate channel weights $\omega_{c}$ and $s_{c}$, where $\omega_{c}$ signifies the weight of channel c, and $s_{c}$ is a scalar serving as the bias term that can be regarded as a learned parameter:

(10) $$z_{c}=f\left(w_{2}f\left(w_{1}\alpha_{c}+b_{1}\right)+b_{2}\right)$$

(11) $$\omega_{c}=\frac{e^{z_{c}}}{\sum_{c^{\prime }=1}^{c}e^{z_{c^{\prime }}}},s_{c}=\left(\gamma -1\right)z_{c}+1$$

In this context, γ serves as a hyperparameter that governs the degree of scaling of the generated weight $w_{c}\in concerning$t to the input feature map and aids in expediting convergence.

### 4.3. ArcFace Loss

In the classification task of single-person gait, where the differences in motion waveforms are not significant, the network needs to effectively differentiate these visually similar images. While the cross-entropy loss function is commonly used in traditional classification tasks, it may require a larger number of iterations to converge in this particular task. Additive angular margin loss, also named ArcFace loss, offers a comparative advantage as it enables faster convergence. 

ArcFace loss is a loss function used to enhance the boundaries of the feature embedding space, primarily used in tasks such as face recognition and feature representation learning [26]. Through experiments, we have observed its potential in multi-classification tasks, as it introduces angle margin parameters and cosine boundaries to compare the cosine similarity between feature vectors and weight vectors with the angular differences between labels. This results in a tighter clustering of feature vectors within the same category and a more dispersed distribution of feature vectors across different categories.

The most widely used classification loss function, soft-max loss, is presented as $L_{1}$.

(12) $$L_{1}=-log\frac{e^{W_{y_{i}}^{T}x_{i}+b_{y_{i}}}}{\sum_{j=1}^{N}e^{W_{j}^{T}x_{i}+b_{j}}}$$

In this context, $x_{i}\in R^{d}$ represents the deep feature of the *i*th sample and $y_{i}$ represents the category of that sample. The feature dimension is temporarily set to 512. $W_{j}\in R^{d}$ represents the weight of the *j*th column in $W\in R^{d\times N}$, and $b_{j}$ is the bias term. However, this soft-max loss does not explicitly optimize feature embeddings to enhance high similarity among intra-class samples and differences among inter-class samples. This can lead to confusion between similar behaviors, such as descending a slope and stepping down a staircase when performing gait recognition. To address this, we consider $W_{j}^{T}x_{i}=\left\|W_{j}\right\|\left\|x_{i}\right\|cos\theta_{j}$, where $\theta_{j}$ is the angle between the weight $W_{j}$ and feature $x_{i}$. By fixing the weights to 1 and normalizing the features with $l_{2}$ regularization and rescaling them to s, the prediction result becomes dependent on the angle between the features and weights. Therefore, the learned embedded features are distributed on a hypersphere with a radius of s. So, the loss function can be represented by $L_{2}$.

(13) $$L_{2}=-\frac{1}{N}\sum_{i=1}^{N}log\frac{e^{s\left(\cos\theta_{y_{i}}\right)}}{e^{s\left(\cos\theta_{y_{i}}\right)}+\sum_{j=1,j\neq y_{i}}^{n}e^{scos\theta_{j}}}$$

To simultaneously increase inter-class compactness and inter-class differences, an additional angular margin penalty m is applied between features and weights. The calculation formula for the AM loss function is $L_{3}$.

(14) $$L_{3}=-\frac{1}{N}\sum_{i=1}^{N}log\frac{e^{s\left(\cos\left(\theta_{y_{i}}+m\right)\right)}}{e^{s\left(\cos\left(\theta_{y_{i}}+m\right)\right)}+\sum_{j=1,j\neq y_{i}}^{n}e^{scos\theta_{j}}}$$

where *L* represents the loss function, s denotes the scaling factor, $\theta_{y_{i}}$ represents the angle between the feature vector and weights of sample *i*, $y_{i}$ represents the authentic category of the sample, and m is the angular interval parameter. 

The objective of angular margin loss during training is to minimize this loss function. It achieves this by optimizing the boundaries of the feature embedding space, enhancing the separability of feature vectors, and ultimately improving the performance of classification.

## 5. Experiments and Results

### 5.1. Experimental Environment

**Figure 6:**
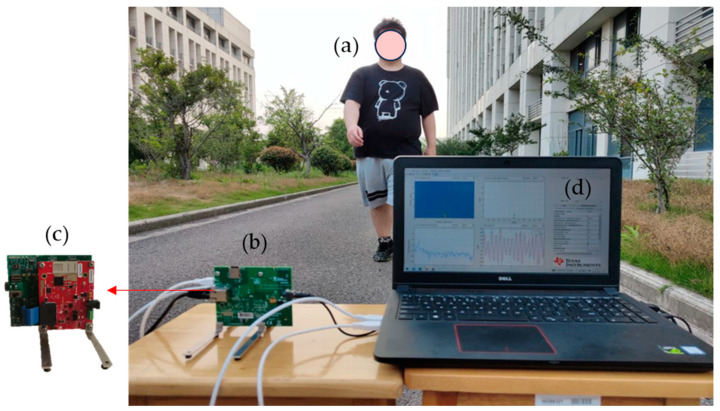
Experiment scene of data collection. (**a**) represents the human target, (**b**) is the AWR1642 radar and DCA1000EVM data acquisition board, (**c**) is the front of the radar and data acquisition board, and (**d**) mmwave studio is the control software of PC.

The experimental data were collected outdoors between buildings on the campus of Nantong University in May 2023. The experiment utilized the AWR1642 radar and DAC1000EVM for data acquisition, both produced by Texas Instruments. The AWR1642 is a multi-channel radar sensor, while the DAC1000EVM is an acquisition card that interfaces with the AWR1642, allowing users to transmit digital intermediate frequency data to a computer via Ethernet. As shown in Figure 6, the experiment took place on a closed driving path within a park, with the FMCW radar positioned facing the direction of the target. In this study, we explored the optimal structure of the proposed network and conducted ablation experiments using our own dataset, which includes seven types of human behaviors. The experiment involved five participants, each with different heights, weights, and ages. The self-built dataset adopted a relatively simple activity acquisition mode, with the movement range for walking and running being approximately 10 m. Considering actions such as descending stairs and slopes require a complete cycle, the duration of each sample data was set to 4 s. During the experiment, to ensure the diversity of the dataset, each participant acted according to their habits, resulting in a total of 700 Doppler sequence groups. In the experiment, we set the system parameters as shown in Table 1.

The model was trained and tested on a computer equipped with a 2.9GHz Intel Core i5-10500F processor, NVIDIA GeForce RTX3060ti GPU, and the Windows 10 operating system. The program is designed using open source software Python 3.7 and PyTorch 12.1.

### 5.2. Network Comparison

In each training session, we randomly selected 16 instances for each task. Within the inner loop, the step size for updates was set to 5, and the inner learning rate, p, was set to 0.01. In the outer loop, we utilized the Adam optimizer with a learning rate λ of 0.0001 and a meta-batch size of 3. The collected dataset was divided into training, validation, and testing sets for evaluation purposes.

Due to positive feedback between layers, the ability to represent features can be amplified during training. Deepening the network can enhance nonlinear expression capabilities, enabling more complex feature fitting. The width of each layer, that is, the number of convolution kernels, determines the richness of captured features in each layer, which is related to the difficulty of network optimization. However, blindly deepening the network can lead to optimization difficulties, performance saturation, and degradation of shallow learning abilities. Similarly, exceeding the appropriate width can reduce network efficiency due to redundant feature extraction. Therefore, a ResNet18 network with relatively few layers was chosen for the inner network. The smaller number of network layers and the stable residual structure helped the MAML network better constrain the inner loop.

In the MAML network framework, due to insufficient constraints within meta-learning, its stability was poor during runtime. Therefore, when optimizing the MAML network, the main consideration was the constraint problem of inner loop optimization. At this time, a stricter inner loop loss helped the network better obtain generalizable parameters during the inner loop and better adapt to gait datasets in small sample scenarios.

AS shown in Table 2, the optimized AS-MAML model performs better on the data set. Compared to other base networks, AS-MAML showed significant improvements in both accuracy at the 95% confidence interval and MAP95 accuracy. With the method proposed in this paper, a significant improvement in the MAP95 accuracy of the model can be observed. Furthermore, by optimizing the internal loss function using the ArcFace loss function, our 95% confidence interval (CI) improved by 3.2%. Because training the network under the MAML framework is to train better parameters for the network, rather than expanding or modifying the structure of the network, the parameter amount and inference time of AS-MAML were not greatly affected. These results show that our proposed MAML loss optimization effectively reduced model overfitting to small samples of FMCW radar micro-Doppler images and enhanced the generalization performance of query samples.

### 5.3. Ablation Experiment

Furthermore, we conducted a thermolysis experiment to examine the effects of the loss function and attention mechanism. We classified the results into the following four categories and compared them based on 95% confidence accuracy and MAP95:

(a) MAML: where the internal training utilized cross-entropy loss through pure MAML training.
(b) MAML and ArcFace loss: inner loop optimization only, without employing attention mechanism.
(c) MAML and SE: utilizing both cross-entropy loss and the SE-attention mechanism.
(d) AS-MAML: our proposed optimized network.

**Figure 7:**
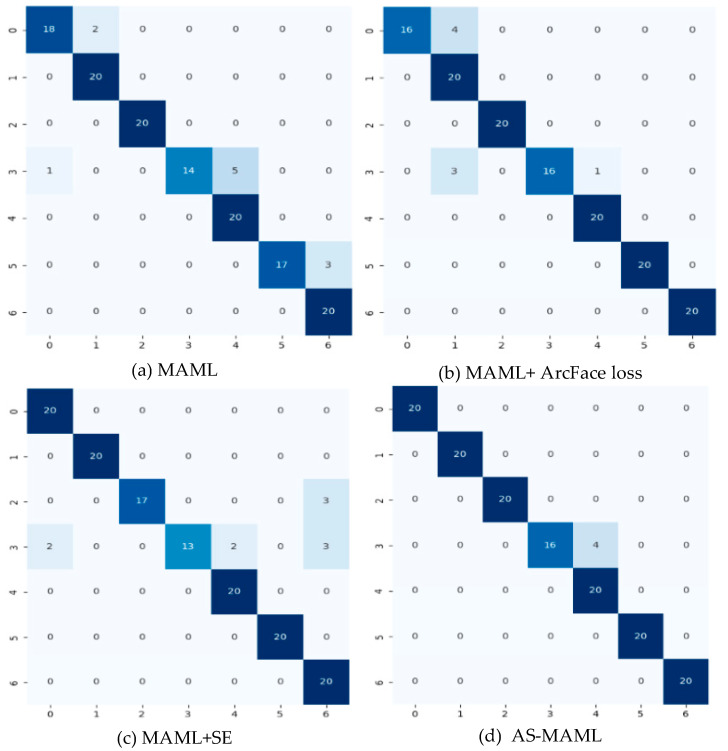
Confusion matrix for ablation experiment. (**a**–**d**) refer to the four ablation experiment results mentioned in the above text, respectively.

In terms of the ablation experiment, our comparative findings are illustrated in Table 3. In contrast to MAML, the optimized MAML showcased notably superior performance on the Res18 network. This observation signifies that the ArcFace loss, along with the SE-attention mechanism, effectively addressed the issue of overfitting during the internal optimization of the model. Moreover, the MAML that incorporates both optimizations concurrently achieved the most exceptional classification outcomes. Since the shot quantity set for each iteration of the inner loop was 10, the accuracy in the training results was relatively high. However, as shown in Figure 7, there was a significant difference with regard to MAP95.

### 5.4. T-SNE Visual Clustering Degree Analysis

**Figure 8:**
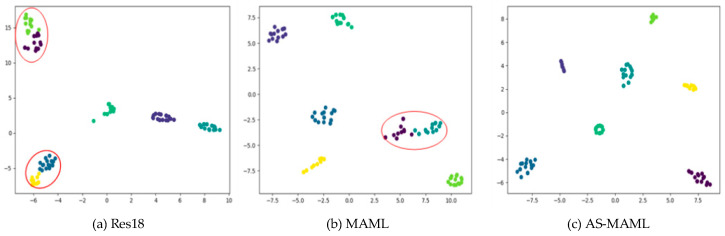
T-SNE visualization results. The clustering effect of (**c**) AS-MAML is significantly better than that of (**a**) Res18 and (**b**) MAML, as the distances between categories are too close, which are circled in red.

Figure 8 shows the T-SNE visualization of different networks, where Figure 8a represents the original Res18 network, Figure 8b represents MAML, and Figure 8c represents AS-MAML. We demonstrated the effectiveness of our method by visualizing the feature distribution of the test. In the figure, we can see that compared with the basic network Res18 and MAML, our method reduced the dispersion of features in each class and had good intra-class aggregation and inter-class separability.

### 5.5. Comparison of Data Augmentation Methods

**Figure 9:**
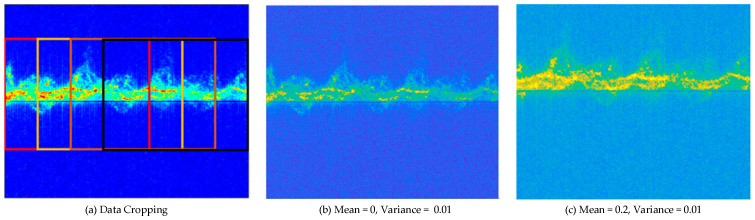
Schematic diagram of image enhancement.

In small sample training, apart from the MAML method mentioned in this article, there are also other approaches, the most common being data augmentation. There are various methods of data augmentation, but cropping or adding noise in time-related spectrograms are commonly used techniques. Specific methods can be seen in Figure 9. The data set is expanded by data enhancement in (a), and the data is enhanced by Gaussian kernel with mean 0 and variance 0.01 and mean 0.2 and variance 0.01 respectively in (b) and (c).

By employing image enhancement techniques, the dataset can be expanded eightfold, providing sufficient data for deep learning training. The internal network of the previous AS-MAML was still used for the training of the network, but instead of using the MAML framework, only conventional network training was performed. During training, the learning rate was set to 0.01, the optimizer was Adam, and the model was trained for 50 epochs. The training environment remained consistent with that described earlier. 

**Figure 10:**
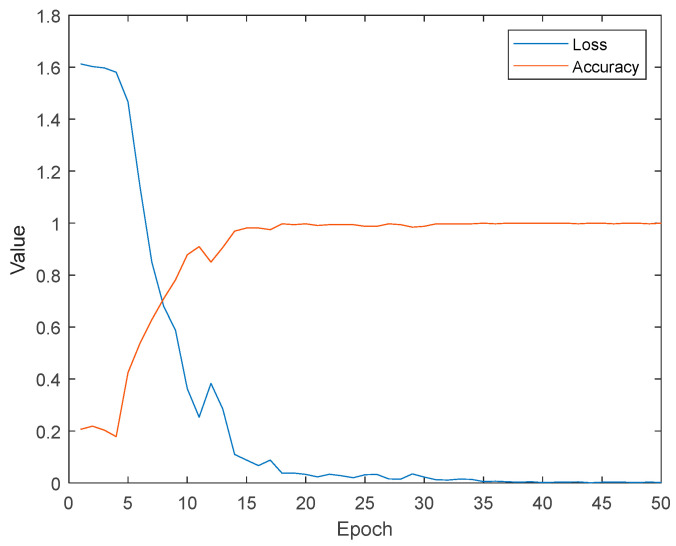
The training loss function and accuracy curves.

The training loss function and accuracy curves are presented in Figure 10.

It can be observed that after training the network 15 times, the accuracy on the training set reached 100%, and both the loss and accuracy remained relatively stable during subsequent training sessions. This indicates that commonly used data augmentation methods can also achieve high classification accuracy. When validating the network on the test set, the obtained accuracy was 92.3%, but the MAP95 result was only 51.2%, indicating that overfitting occurred during network training. During the process of training with a limited number of samples, overfitting is a common and easily occurring problem. It can lead to the algorithm’s inability to adapt to unseen datasets and decrease its generalization ability, resulting in the inability to accurately identify the poses of pedestrians outside the dataset during practical use. Therefore, it is crucial to avoid the occurrence of overfitting during the training process.

### 5.6. Open-Source Dataset Comparison

To validate our network, an open-source dataset, DIAT-μRadHAR [30], was selected, which contained samples of six human activities: army crawling, army jogging, jumping while holding a gun, army marching, boxing, and stone-pelting/grenades-throwing. When configuring the network, the optimizer was set to Adam with a learning rate of 0.0001, and a total of 100 epochs were trained. Due to the significant inter-class differences in this dataset, employing basic convolutional neural networks such as VGG16 or ResNet18 yielded excellent training results. After 100 training iterations, the model was already well-fitted, maintaining an accuracy of over 99%. To further enhance the validation of our network, we designed the dataset by compressing images and sampling data through random cropping in the temporal dimension. Such approaches make the image dataset features less distinct and more challenging to validate.

**Figure 11:**
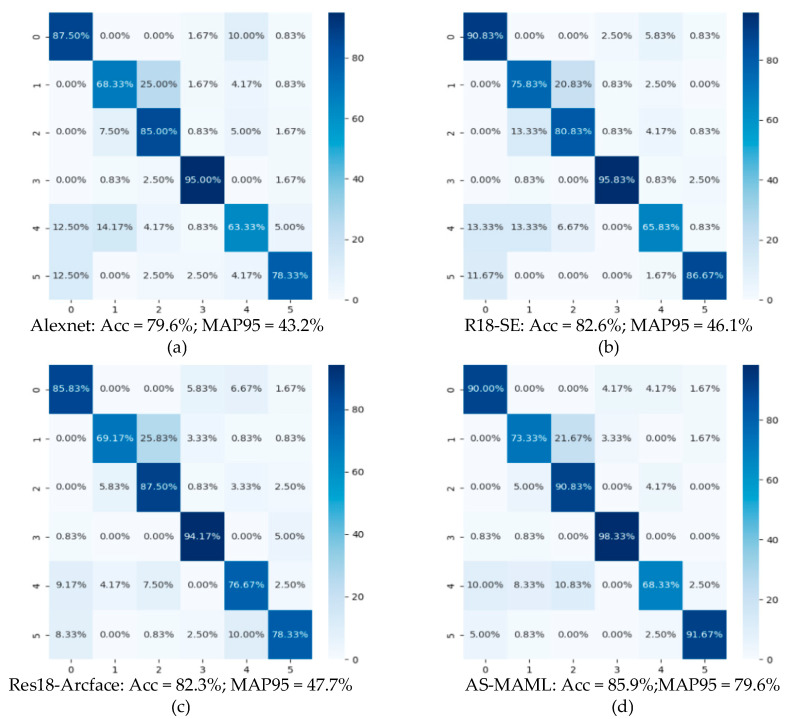
Confusion matrices for the proposed and compared networks.

From Figure 11, it can be observed that our network outperformed some basic convolutional neural networks in terms of training results. Not only did it achieve the highest accuracy, but also the MAP95 parameters were the most outstanding among the aforementioned networks. This fully validates the effectiveness of our network.

### 5.7. Classification of Different Walking Modes

**Figure 12:**
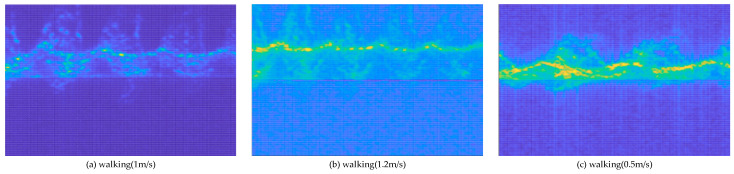
Micro-Doppler images with three walking speeds.

Taking into account different walking habits and each person’s special circumstances, the walking speed may be different. There are differences in the walking speed of young, middle-aged, and old people, which leads to differences in the spectral map generated by FMCW radar. Therefore, it is also important to classify and identify pedestrians at different speeds. As shown in Figure 12, we collected data at three different traveling speeds. The first group is normal walking with a speed of about 1 m/s, the second group is fast walking with a speed of 1.2 m/s, and the third group is slow walking with a speed of about 0.5 m/s.

**Figure 13:**
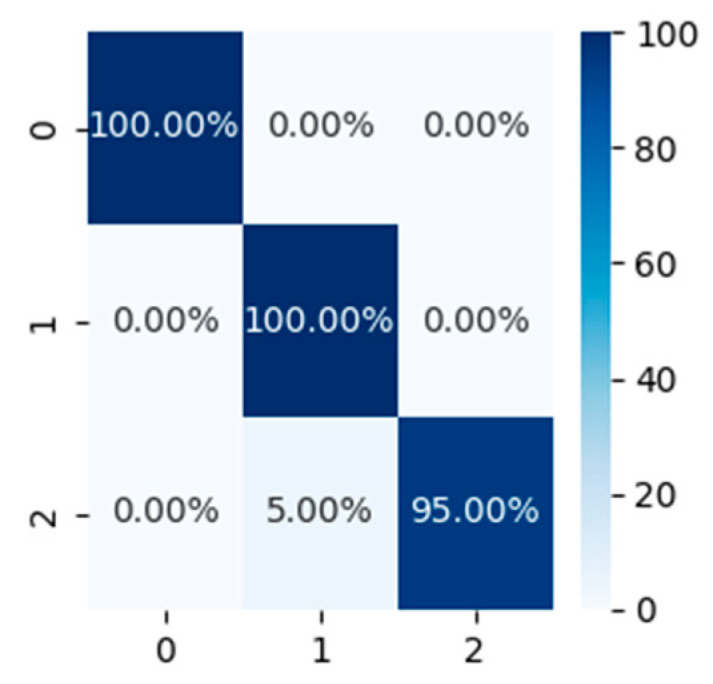
Confusion matrix for classes with three walking speeds.

The classification results are shown in Figure 13. At different walking speeds, the pedestrian’s main speed and the swing speed of the limbs change, resulting in different spectrum diagrams, which can be used to classify. In this group of classifications, the classification accuracy reached 98.3%, which also reflects the effectiveness of classification under different walking speeds.

**Figure 14:**
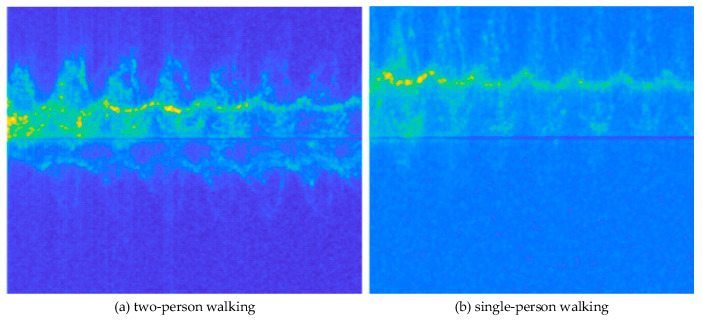
Micro-Doppler images of two-person walking and single-person walking.

We also conducted experiments on the discrimination between the two-person walking and single-person walking situations. We compared the difference between a single-person walking and two-person walking in two ways, that is, walking in the same directions and opposite directions: (1) when two people are walking in the same direction, their stride frequencies will overlap, causing the signal intensity of the spectrum within the same category to increase, which is easily consistent with the spectrum of a single person walking. In this situation, multiple targets can be distinguished based on characteristics such as the target azimuth angle and distance. (2) When two people walk in opposite directions, people can be identified by Doppler frequency shift without acquiring the target azimuth angle or distance. This is because the spectrograms of two people opposite on walks are quite different, which can realize the classification. The Doppler spectrogram of one example of the two-person and single-person walks is shown in Figure 14. Where (a) is the spectral diagram of two people walking opposite each other, and (b) is the spectral diagram of one person walking.

**Figure 15:**
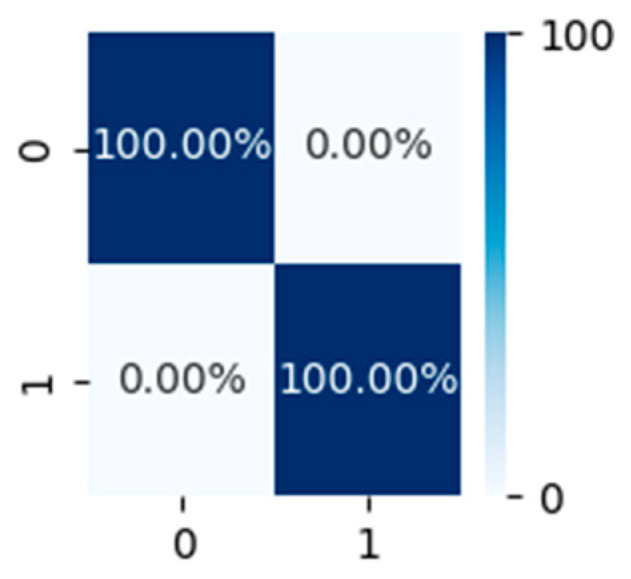
Confusion matrix for two-person and single-person walking.

From Figure 15, we can observe that when two people walk in opposite directions, the two-person walking and single-person walking situations can be clearly distinguished. This is because there is a big difference between the spectrogram of two-person walking in opposite directions and the spectrogram of single-person walking. The classification results perform well, and the two different walking modes can be accurately identified in the binary classification task.

## 6. Conclusions

We propose an AS-MAML method for pedestrian pose detection using FMCW radar. By utilizing micro-Doppler images of FMCW radar, pedestrian gait recognition is achieved. By using the channel attention mechanism in the inner loop network to constrain the inner loop and by adding an angle margin loss function to expand the gap between classes and reduce the distance within the class, it ensures that similar samples can be more effectively restricted when updating parameters. Finally, the AS-MAML network with the constrained inner loop was used for training on the self-sampling dataset and verified on the open-source dataset DIAT-*μ*RadHAR. According to the experimental results, compared with the traditional CNN network, our network performed better in small samples. It can show a greater advantage on the dataset, reaching 94.3%, and it also has a classification accuracy of 85.9% on the randomly sampled open-source dataset. However, it is worth noting that this method is currently in the experimental stage, and our plans include integrating it with outdoor autonomous driving or surveillance functions and continuing to develop multi-sensor pedestrian recognition. 

## Figures and Tables

**Table 1:** System parameters.

Parameter	Symbol	Value
Number of ADC samples	NTS	256
Number of Chirps	$N_{c}$	128
Chirps Time	$T_{c}$	32 μs
Number of Transmit Antennas	$N_{TX}$	2
Number of Receive Antennas	$N_{RX}$	4
Total Bandwidth	B	1.8 GHz
Frame Time	$T_{f}$	40 ms

**Table 2:** The classification results of different models.

Model	95% CI	MAP95	Parameters	Inference Time/Sample (ms)
Resnet18 [27]	91.3	65.7	4,728,774	4.10
PCA [14]	76.6	43.7	53,191	5.13
AlexNet [28]	71.4	47.0	57,017,031	4.46
VGG16 [29]	87.1	64.3	134,292,422	16.01
AS-MAML	94.5	93.7	4,730,311	4.11

**Table 3:** Classification results for different positions of attention modules.

	ArcFace Loss	SE-Attention	95% CI	MAP95
(a)			92.1	79.6
(b)	√		93.7	86.2
(c)		√	92.8	89.7
(d)	√	√	94.5	93.7

## Data Availability

Data are available on request from the authors.
